# Successful Percutaneous Coronary Intervention in Chronic Total Occlusion after Coronary Perforation

**DOI:** 10.1155/2023/6640439

**Published:** 2023-08-30

**Authors:** Luis A. Areiza, Juan F. Rodriguez

**Affiliations:** Department of Interventional Cardiology, HUM Mederi, Bogota, Colombia

## Abstract

Coronary chronic total occlusions are challenging lesions with high rates of complications related to percutaneous intervention. We describe a successful angioplasty in a patient with a recent coronary perforation, using multiple techniques, such as stick and swap with Stingray, subintimal transcatheter withdrawal, and investment.

## 1. Introduction

Percutaneous coronary intervention (PCI) for chronic total occlusion (CTO) is an available alternative in cases of symptomatic disease. Coronary artery perforation is an infrequent complication of non-CTO PCI, however, the incidence may be higher for CTO interventions. Defining the timing for a second attempt of PCI after coronary perforation is challenging, therefore, understanding the physiopathological aspects of these lesions is the cornerstone of a successful angioplasty.

### 1.1. Case Report

A 61-year-old male during preoperative studies for ophthalmologic surgery, presented dyspnea with moderate physical activity, New York Heart Association functional class II, and chest pain. Physical examination demonstrated normal heart auscultation, no signs of fluid overload, and normal vital signs. Past medical history was relevant for childhood nephritic syndrome, and family medical history is negative for cardiovascular disease. The patient had smoked half a pack of cigarettes a day for 10 years but quit smoking for 12 months. He did not have any recent hospital admissions.

A transthoracic echocardiogram showed concentric left ventricular hypertrophy and no other abnormalities. Myocardial perfusion imaging demonstrated inferior wall ischemia of 10%.

With these results, a coronary arteriography was performed showing severe coronary disease of the left anterior descending (LAD) artery and a CTO in the middle segment of the right coronary artery (RCA).

**Figure 1:**
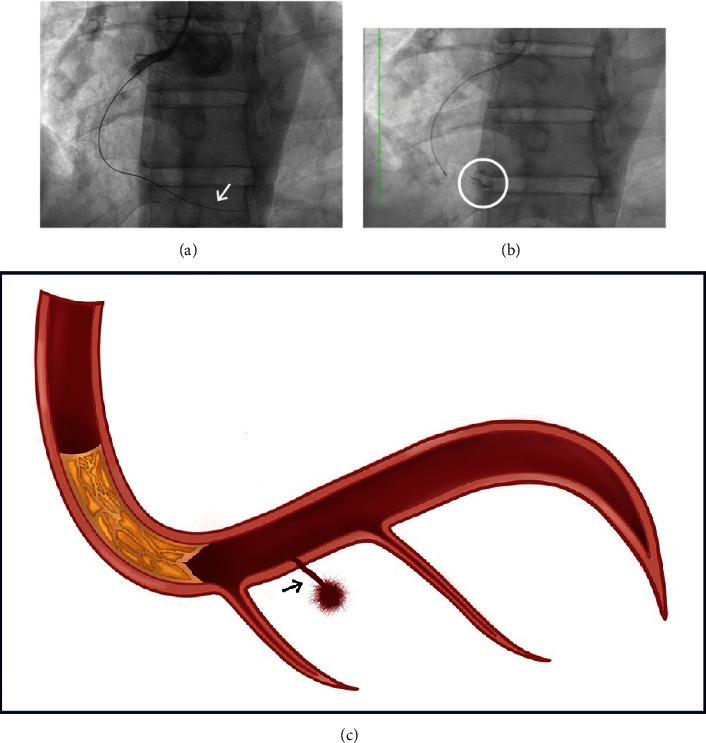
RCA perforation. Fluoroscopy of the RCA showing. (a) Wire outside the vessel architecture (arrow). (b) Contrast extravasation (circle). (c) Illustration of RCA perforation (arrow).

Taking into consideration the symptomatic burden and viability studies results, the Interventional Cardiology team of another institution decided to attempt CTO PCI and LAD PCI, beginning with the CTO, which was the most complex lesion, using an anterograde approach. During the procedure, the distal part of the wire was observed outside the RCA architecture, and contrast extravasation occurred, leading to the suspension of the procedure (Figure 1). The coronary perforation affected all layers of the vessel and was categorized as Ellis 2, with contrast extravasation limited to the myocardium and an orifice of less than 1 mm. The patient presented stable vital signs, and conservative management was successful leading to hospital discharge 3 days after the procedure.

One month later, the patient was admitted to our institution presenting unstable angina, and we performed PCI over the LAD. At that time, the main challenge was to define the pertinence and timing for a potential second attempt for CTO PCI. As a result of the initial coronary perforation, the vessel was undergoing a healing process, and it was crucial to complete all phases to minimize the risk of further complications.

Since the patient continued to experience coronary symptoms despite invasive management of other coronary lesions and optimal medical therapy, we considered it necessary to proceed with a second attempt for CTO angioplasty. To reduce the risk of re-perforation during PCI, restoration of the original structure of the vessel was required and achieved through the healing process of hemostasis, proliferation, and remodeling. Based on the latter, a CTO PCI attempt using coronary computed tomography (CCT) for planning purposes was performed 60 days after perforation.

Considering an occlusion length ≥20 mm, a blunt entry shape, and evidence of calcification, a Japan-CTO score of 3 points was calculated. CCT confirmed that the vessel architecture was restored, excluding abnormalities, such as coronary fistula, pseudo-aneurysms, or true aneurysms.

It should be noted that at that moment the patient continued to experience coronary symptoms.

**Figure 2:**
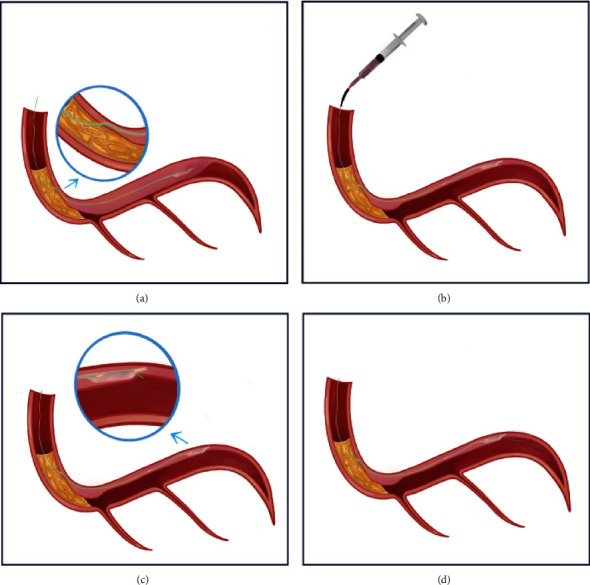
Wire entering extraplaque space, subintimal transcatheter withdrawal technique, and reentry to the posterolateral artery true lumen. (a) False lumen hematoma before subintimal transcatheter withdrawal technique. (b) Subintimal transcatheter withdrawal technique. Aspiration is performed by decompressing the hematoma through the STINGRAY™ and allowing re-expansion of the distal true lumen. (c) Reentry to the posterolateral artery true lumen using the stick and swap. (d) Distal position of the wire in the posterolateral artery (PLA) lumen.

A primary retrograde approach was performed reaching no further than the extraplaque middle segment of the RCA. Hence, the approach was changed to antegrade. The RCA was engaged using an 8 Fr. AL 1 catheter (Cordis Corporation, Miami, FL, USA), the proximal cap was punctured with a stiff HORNET™ 14 wire (Boston Scientific Corporation, Marlborough, MA, USA), and the CTO body traversed with a FIGHTER™ wire (Boston Scientific Corporation) over a MAMBA™ Flex microcatheter wire (Boston Scientific Corporation), but the distal true lumen was not reached. FIGHTER™ wire was advanced to the proximal posterolateral artery (PLA), entering the false lumen, and then a STINGRAY™ system (Boston Scientific Corporation) was delivered to drain 5 cc from the subintimal hematoma with the STRAW (subintimal transcatheter withdrawal) technique. Subsequently, a reentry to the PLA true lumen was achieved using the stick and swap technique (Figure 2).

The intraluminal distal position of the wire was confirmed. To preserve the side branch (posterior descending artery), we performed an investment procedure (subintimal plaque modification) with conventional balloon angioplasty. Based on current evidence regarding the timing for deferred stenting, we wait an additional 60 days for stent implantation, aiming for dissection plane healing.

**Figure 3:**
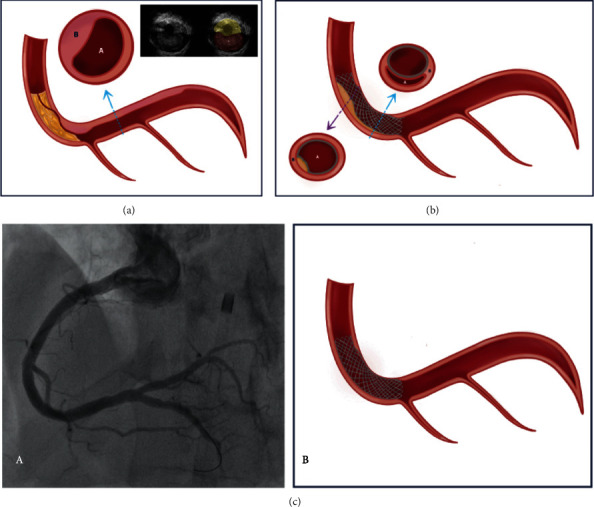
Pre-stent implantation arteriography illustration and final angiographic result. (a) Lesion in the middle segment of the RCA, with signs of dissection and a microchannel filling the distal segment of the artery. Blue arrow: Cross-section showing true lumen (A) and false lumen (B). Intravascular ultrasound demarcated false lumen (FL) true lumen (TL) and wire (arrow). (b) Purple arrow: proximal segment of the stent in the true lumen (A). Blue arrow: distal segment of the stent in the false lumen (B). (c) Final angiographic result (A) and illustration (B), following successful angioplasty with stent implantation.

After this period, coronary angiography showed signs of dissection and a microchannel filling the distal RCA. Conventional balloon angioplasty and drug-eluting stent implantation were performed. Control angiography demonstrated a satisfactory result (Figure 3). Intravascular ultrasound confirmed the appropriate positioning of the stent without signs of dissection.

In the follow-up appointment, four months after the last PCI the patient denied chest pain and dyspnea at rest. He tolerated moderate physical activity without symptoms. No new admissions or coronary interventions were required.

## 2. Discussion

PCI for CTO has demonstrated relief in symptom burden and a reduction in major adverse cardiovascular events [1]. However, some complications may occur more frequently compared with non-CTO PCI.

Coronary perforation can be categorized according to Ellis's classification. Type I perforation refers to an extraluminal crater without extravasation, type II to a pericardial or myocardial blush without contrast jet extravasation, and type III to an extravasation through ≥1 mm perforation [2]. This is a relatively common complication of PCI for CTOs. El Sabbagh et al. [3] reported a risk of 4.3%, which can be attributed to the complexity of the procedure and the vulnerability of highly calcified vessels.

**Figure 4:**
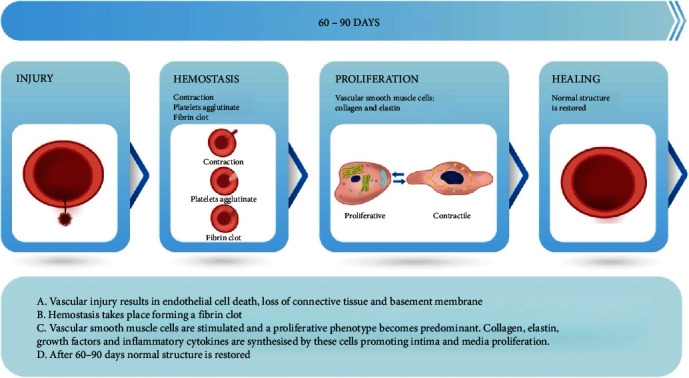
Vessel healing. Vessel healing process: description and illustration of injury, hemostasis, proliferation, and healing. Adapted from Bacakova et al. [4].

In our patient, the presence of chest pain and positive myocardial perfusion imaging were clear indications to perform CTO PCI. After the coronary artery perforation Ellis type II, treated with conservative management, the next objective was to accurately determine the time for a second-time PCI. We decided to wait 60 days before performing a new attempt, based on the physiopathological process of vessel healing [4] (Figure 4). An earlier intervention might have increased the risk of complications, such as coronary perforation, in a not completely healed vessel.

Choosing a technique for CTO PCI requires the analysis of multiple variables, such as proximal cap characterization, distal vessel quality, lesion length, and collateral circulation. It is also important to determine the optimal timing for transitioning between available strategies, as this can help reduce the risk of complications associated with the procedure. Wu et al. [5] presented an algorithm for the CTO PCI crossing procedure. In our case, the complexity of the lesion resulted in a failed retrograde approach with a posterior antegrade dissection and reentry. Ultimately, to preserve flow in the side branch (posterior descending artery), we performed subintimal plaque modification as an “investment procedure.”

Subintimal plaque modification consists of a technique, in which balloon angioplasty is performed through the subintimal space aiming for anterograde flow. This procedure requires the healing of dissection planes and hematoma before stent implantation. Timing for deferred stenting in different studies varies between 1.5 and 4 months [6]. This is a variation of subintimal tracking and reentry, which have high success rates, but also have a significant risk of re-stenosis [7] and can compromise flow through side branches.

Follow-up 2.5 ± 0.3 months after subintimal plaque modification, showed that 70% of the patients preserve TIMI 3 flow of the dissected artery [8]. Xenogiannis et al. [9] reported outcomes of subintimal plaque modification in CTO, finding an improvement in successful rates with an acceptable risk of major adverse cardiovascular events. In this study, waiting at least 2 months before stent implantation was associated with higher technical success.

## 3. Conclusion

Defining the timing of a second attempt of coronary angioplasty after coronary perforation is challenging and current data is limited in this area. Waiting enough time to allow complete vessel healing may be necessary to make new PCI attempts. In our patient, previous iatrogenic complications did not represent a limitation to a new successful intervention.
